# Inter-individual variation in morphine clearance in children

**DOI:** 10.1007/s00228-015-1843-x

**Published:** 2015-04-08

**Authors:** Mohammed I. Altamimi, Imti Choonara, Helen Sammons

**Affiliations:** Academic Division of Child Health, University of Nottingham, Derbyshire Children’s Hospital, Derby, DE22 3DT UK

**Keywords:** Morphine, Pharmacokinetics, Clearance, Children, Variation

## Abstract

**Objectives:**

The aim of the study was to determine the extent of inter-individual variation in clearance of intravenous morphine in children and to establish which factors are responsible for this variation.

**Methods:**

A systematic literature review was performed to identify papers describing the clearance of morphine in children. The following databases were searched: Medline, Embase, International Pharmaceutical Abstracts, CINAHL, and Cochrane library. From the papers, the range in plasma clearance and the coefficient of variation (CV) in plasma clearance were determined.

**Results:**

Twenty-eight studies were identified. After quality assessment, 20 studies were included. Only 10 studies gave clearance values for individual patients. The majority of the studies were in critically ill patients. Inter-individual variability of morphine clearance was observed in all age groups, but greatest in critically ill neonates (both preterm and term) and infants. In critically ill patients, the CV was 16–9 7 % in preterm neonates, 24–87 % in term neonates, 35 and 134 % in infants, 39 and 55 % in children, and 74 % in adolescents. The CV was 37 and 44 % respectively in non-critically ill neonates and infants. The mean clearance was higher in children (32 and 52 ml min^-1^ kg^-1^) than in neonates (2 to 16 ml min^-1^ kg^-1^).

**Conclusions:**

Large inter-individual variation was seen in morphine clearance values in critically ill neonates and infants.

**Electronic supplementary material:**

The online version of this article (doi:10.1007/s00228-015-1843-x) contains supplementary material, which is available to authorized users.

## Introduction

Morphine is a naturally occurring opioid alkaloid. It is the first choice analgesic for severe pain and can be used for preoperative sedation. Morphine can be administered via different routes intravenously (IV), intramuscularly (IM), subcutaneously (SC), orally, and rectally. There are two major formulations of oral morphine: immediate release, which has extensive inter-individual variation for bioavailability and controlled release morphine, which is less variable. Bioavailability is approximately 30–40 % [1].

Dosing varies according to the age, route of administration, and is based on body weight [2–4]. Due to patients’ varied response to pain, morphine dose is usually titrated according to clinical response. Paediatric dosing for drugs is sometimes determined from adult pharmacokinetic studies. However, results from adults are difficult to extrapolate to children, because the physiological makeup of the two age groups is different. Pharmacokinetic studies in children therefore help to ensure that the appropriate drug dose is administered. Doses are usually calculated from mean pharmacokinetic values. There is however often significant variability in pharmacokinetics due to factors such as age [5], weight [6], disease [7], and ethnicity/genotype [8]. Recent advances in research has resulted in the development of PK–models to ensure the appropriate individualisation of dosing in children [9].

We have previously evaluated inter-individual variation in the clearance of midazolam [10]. We wished to explore inter-individual variation in morphine clearance in paediatric patients and we therefore performed a systematic review of pharmacokinetic studies in paediatric patients involving morphine. The metabolite morphine-6-glucuronide (M6G) is more potent as an analgesic than morphine itself. We therefore also looked at M6G to morphine ratios.

## Methods

A systematic literature search was performed to identify all papers describing the clearance of morphine in children. The following databases were utilised; MEDLINE (1946 to May 2013), EMBASE (1974 to May 2013), International Pharmaceutical Abstracts (1970 to April 2013), CINAHL, and Cochrane Library. The databases were searched separately and combined together to remove duplications. The search strategy included all languages and involved the keywords “morphine AND “child*” OR “p*ediatric* OR “infant*” OR “new-born*” OR “neonate*” OR “adolescent*” [11] AND “pharmacokinetic*” OR “clearance” OR “half-life” OR “absorption” OR “distribution” OR “metabolism” OR “elimination” OR *”.

Inclusion criteria were original research studies assessing the pharmacokinetics of morphine in children up to the age of 18 years. We excluded the following: review articles, editorials, conference abstracts, studies in adults aged 18 years and over, and studies that involved adults and paediatric patients where the paediatric data was not presented separately. Studies in which morphine was not administered intravenously were also excluded.

Data such as, number of patients, ethnicity, dose, and clearance were extracted. The mean/median clearance, as well as both the minimum and maximum clearance values were noted. The variation ratio was calculated from the range of clearance (maximum clearance divided by the minimum clearance).The coefficient of variation (CV) was extracted from the paper if given. If individual data was presented, then the CV was calculated using the formula [10, 12].

$$ \mathrm{C}\mathrm{V} = \sqrt{\left(\left({\mathrm{e}\ \mathrm{S}\mathrm{D}}^2\right) - 1\right)} $$

which allows for the fact that clearance is usually log normally distributed in children. If individual data was not available, then CV was estimated by dividing the standard deviation by the mean of clearance, i.e., normal distribution assumed. The variation ratio for M6G/M was calculated by dividing the maximum M6G/M by the minimum M6G/M. Patients were divided into two groups: (1) critically ill if they were in an intensive care unit and (2) non-critically ill which included other groups. We also contacted original authors by email if their paper did not give the full range of clearance values but gave mean clearance values.

## Results

**Fig. 1:**
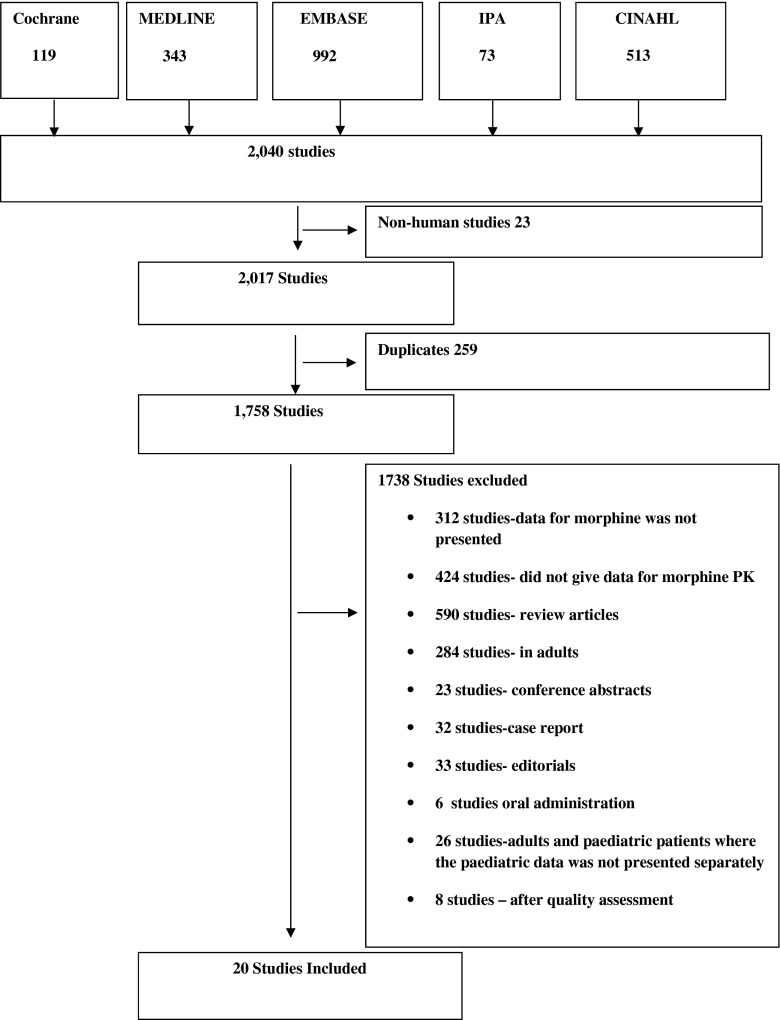
Flow chart of the search performed

A total of 2040 articles were identified, but only 28 articles (studies) met the inclusion criteria [5, 7, 13–38] (Fig. 1).

Eight studies were excluded after quality assessment (Supplementary table 3) [19, 23, 29–31, 35–37]. For one study, some of the data were excluded [5]. A total of 20 studies were included after quality assessment.

The majority of the studies (19) used non-compartmental methods. Only one used a two-compartmental model [34]. Ethnicity was not described in any study. The CV was not stated in any study. Ten studies provided individual data allowing calculation of CV [5, 7, 13–16, 18, 24, 28, 32]. In six studies, only the standard deviation (SD) was reported, and therefore, CV was estimated, assuming normal distribution [17, 20–22, 26, 34]. Three studies did not report either SD or CV of morphine clearance [25, 27, 33].

All the studies, except two, involved critically ill patients [5, 27]. Thirteen studies reported morphine clearance in 228 critically ill neonates (Table 1) [5, 13–18, 20–22, 24–26].

**Table 1:** Morphine clearance in critically ill neonates

Age group	Number of patients	Range of weight (kg)	Mean clearance (ml min^-1^ kg^-1^)	SD	Coefficient of variation (%)	Range of clearance (ml min^-1^ kg^-1^)	Variation ratio in clearance	Comments	Study
Preterm neonates	9	0.9–2	2	0.4	16	2–3	2	Individual data available	Hartley et al 1993 [13]
	8	2–4	5	1.8	47	2–6	3	Individual data available	Mikkelsen et al 1994 [14]
	26	0.7–4	4	1.7	38	2–10	5	Individual data available	Barrett et al 1991 [15]
	10	1-4	3	1.8	96	0.5–7	14	Individual data available	Chay et al 1992 [16]
	31	1^a^	2	1	50	0.8–6	8	–	Saarenmaa et al 2000 [17]
	19	0.7–2	5	3	71	1–14	14	Individual data available	Barrett et al 1996 [18]
	11	2–4	12	9	75	3–35	12	–	Geiduschek et al 1997 [20]
	10^c^ 7^c^	1^b^ 2^b^	3 10	3 4	97 42	NA	NA	–	Bhat et al 1990 [21]
	9^c^ 13^c^ 13^c^	1^b^ 1^b^ 2^b^	2 3 5	1 2 2	47 66 40	NA NA NA	NA NA NA	–	Scott et al 1999 [22]
Term neonates	6	0.5–4	6	2	35	4–10	3	Individual data available	Lynn et al 1987 [24]
	18	2–5	7	NA	NA	3–14	5	–	McRorie et al 1992 [25]
	5	2–4	5	1.8	55	2–7	4	Individual data available	Mikkelsen et al 1994 [14]
	5	3–4	2	1.4	74	0.8–4	5	Individual data available	Chay et al 1992 [16]
	10	1–5	5	4	87	1–13	13	Individual data available	Pokela et al 1993 [5]
	12	2–4	8	2	24	2–39	20	–	Koren et al 1985 [26]
	3	3^b^	16	10	65	NA	NA	–	Bhat et al 1990 [21]
	3	3^b^	8	3	37	NA	NA	–	Scott et al 1999 [22]

*NA* not available

^a^Median

^b^Mean

^c^Two groups of preterm neonates

Details of the administration of morphine and the number of blood samples collected to calculate clearance are given in Supplementary Table 1.

**Fig. 2:**
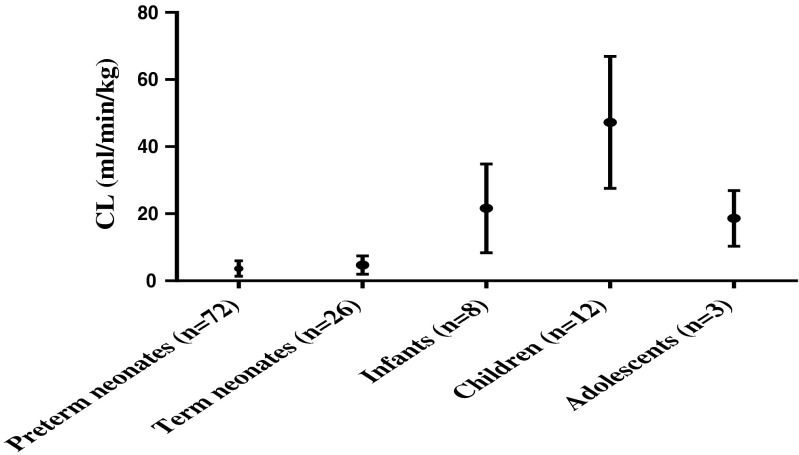
Mean and range of clearance for different patient age groups (for studies with individual data of CL)

Nine of the neonatal studies were in preterm neonates (*n* = 166) with CV ranging from 16 to 97 %. The CV for term neonates (*n* = 62) varied between 24 and 87 %. There was 2–14-fold inter-individual variation of clearance in preterm neonates and 3–20-fold variation in term neonates. The range in clearance is shown in Fig. 2.

There were four studies involving 53 infants (Table 2) [24, 25, 27, 28].

**Table 2:** Morphine clearance in critically ill paediatric patients

Age group	Number of patients	Range of weight (kg)	Mean clearance (ml min^-1^ kg^-1^)	SD	Coefficient of variation (%)	Range of clearance (ml min^-1^ kg^-1^)	Variation ratio in clearance	Comments	Study
Infants	10	2–4	0.7^a^	NA	NA	0.6–1	2	–	Roka et al 2008 [27]
	5	3–6	22	17	134	4–45	11	Individual data available	Olkkola et al 1988 [28]
	3	3–6	24	14	35	13–39	3	Individual data available	Lynn et al 1987 [24]
	35	3–14	NA	NA	NA	6–29	5	–	McRorie et al 1992 [25]
12–11 years	9	13–26	52	18	39	26–76	3	Individual data available	Olkkola et al 1988 [28]
	3	21–40	32	18	55	21–53	3	Individual data available	Nahata et al 1985 [32]
12–18 years	3	50–60	19	8	74	9–24	3	Individual data available	Nahata et al 1985 [32]
1–15 years	10	NA	34	NA	NA	19–58	3	–	Collins et al 1996 [33]
6–19 years	18	NA	36	14	39	6–59	10	–	Dampier et al 1995 [34]
7 months–7 years	8^b^ 8^c^	NA	14 23	5 6	37 25	8–22 18–33	3 2	Individual data available	Dagan et al 1993 [7]

*NA* not available

^a^Median

^b^Fontan repair

^c^Tetralogy of Fallot (TOF)

Two of these studies involved both critically ill and non-critically ill patients (Table 3).

**Table 3:** Morphine clearance in non-critically ill paediatric patients

Age group	Number of patients	Range of weight (kg)	Mean Clearance (ml min^-1^ kg^-1^)	SD	Coefficient of variation (%)	Range of clearance (ml min^-1^ kg^-1^)	Variation ratio in clearance	Comments	Study
Term neonates	10	3–5	10	3.5	37	6-16	3	Individual data available	Pokela et al 1993 [5]
>28 days–23 months	5	4–8	21	8	44	10-31	3	Individual data available	Pokela et al 1993 [5]
	6	2–4	1^a^	NA	NA	0.6-1	2	–	Roka et al 2008 [27]

*NA* not available

^a^Median

One of these studies involved both term neonates and infants with CV of 37 and 44 %, respectively [5]. The other study involving infants did not report the CV. However, variation ratios in these studies were twofold and threefold [5, 27]. The CV of morphine clearance in critically ill infants was 35 and 134 %. The variation ratio in studies involving critically ill infants was between 2- and 11-fold.

Two studies reported clearance in 12 critically ill children [28, 32]. The CV in these studies was 39 and 55 %. These studies gave the full range of clearance for inter-individual children. The variation ratio of clearance was threefold for both studies. Only one study involved adolescents. The four critically ill adolescents had CV of 74 % and variation ratio of threefold [32].

Three studies involved a combination of more than one age group [7, 33, 34]. The highest CV of 39 % was in a study involving patients aged 6–19 years, whilst the lowest CV of 25 % was in patients aged 7 months–7 years. The degree of variability in clearance was between 2- and 10-fold. The coefficients of variation in different age groups are summarised in Table 4.

**Table 4:** Coefficient of variation for morphine clearance in paediatrics

Age	Critically ill (*n*)	Non-critically ill (*n*)
Preterm neonates	16–97 % (166)	–
Term neonates	24–87 % (62)	37 % (10)
Infants	35–134 % (53)	44 % (11)
Children	39–55 % (12)	–
Adolescents	74 % (4)	–

Three studies reported the ratio of M6G to morphine (Supplementary table 2) [18, 33, 38]. The variation ratio in neonates ranged between 4- and 33-fold. One study in infants reported variation ratio of 12-fold.

## Discussion

Inter-individual variation was greatest in critically ill neonates and infants. Inter-individual variation was lowest in non-critically ill patients. The variation ratio in clearance in critically ill neonates ranged from 2- to 20-fold with the coefficient of variation going up to 96 %. In infants, the variation ratio ranged from 2- to 11-fold with the coefficient of variation up to 134 %. In contrast, the variation ratio in non-critically ill patients and critically ill children and adolescents was less than fourfold (with the exception of one study in critically ill children where it was almost 10-fold).

Intravenous morphine is administered as a fixed dose bolus (50 μg/kg in neonates and 100 μg/kg in infants and children up to the age of 12 years [4]. It is then given as an intravenous infusion with a fourfold variation in dosage in neonates (5–20 μg/kg/h) and lower variation in infusion rates in infants (10–30 μg/kg/h) and children (20–30 μg/kg/h). The dose is then titrated according to clinical response. Titration of dose is essential due to the large inter-individual variation in clearance. The considerable variation in M6G/M ratio especially in neonates suggests that the variation in dosage requirements in order to achieve adequate pain relief is far greater than that currently recommended.

The large inter-individual variation in critically ill neonates and infants is similar to our previous study on inter-individual variation in midazolam clearance in children. In contrast, there was, however, less inter-individual variation in critically ill children and adolescents with morphine than with midazolam. Critically ill patients tend to have impaired renal and hepatic function. Hepatic blood flow and hepatocellular function are altered, and consequently, hepatic clearance of morphine can be impaired [39]. Critically ill children are also at risk of acute renal failure. Impairment of morphine clearance prolongs the drug half-life and accumulation of drugs may occur, leading to toxicity [40]. Conditions such as sepsis, major surgery, and use of nephrotoxic drugs can damage the kidneys thereby altering the pharmacokinetic parameters of morphine [41].

In conclusion, large inter-individual variation was seen in morphine clearance values in critically ill neonates and infants.

## Electronic supplementary material

ESM 1 (DOCX 58 kb)

## References

[1] Hand C, Moore R, McQuay H, Allen M, Sear J (1987). Analysis of morphine and its major metabolites by differential radioimmunoassay. Ann Clin Biochem.

[2] Baber N, Pritchard D (2003). Dose estimation for children. Br J Clin Pharmacol.

[3] Anderson BJ, Holford NH (2013). Understanding dosing: children are small adults, neonates are immature children. Arch Dis Child.

[4] Joint Formulary Committee. British national formulary for children (2014) British Medical Association and Royal Pharmaceutical Society,2014, london

[5] Pokela M, Olkkola K, Seppälä T, Koivisto M (1993). Age-related morphine kinetics in infants. Dev Pharmacol Ther.

[6] Knibbe CA, Krekels EH, van den Anker JN, DeJongh J, Santen GW, van Dijk M, Simons SH, van Lingen RA, Jacqz-Aigrain EM, Danhof M (2009). Morphine glucuronidation in preterm neonates, infants and children younger than 3 years. Clin Pharmacokinet.

[7] Dagan O, Klein J, Bohn D, Barker G, Koren G (1993). Morphine pharmacokinetics in children following cardiac surgery: effects of disease and inotropic support. J Cardiothorac Vasc Anesth.

[8] Klepstad P, Dale O, Skorpen F, Borchgrevink PC, Kaasa S (2005). Genetic variability and clinical efficacy of morphine. Acta Anaesthesiol Scand.

[9] Admiraal R, van Kesteren C, Boelens JJ, Bredius RG, Tibboel D, Knibbe CA (2014). Towards evidence-based dosing regimens in children on the basis of population pharmacokinetic pharmacodynamic modelling. Arch Dis Child.

[10] Altamimi MI SH, Choonara I (2014) Inter-individual variation in midazolam clearance in children. Arch Dis Child 0:1-6 10.1136/archdischild-2013-305720 PMC4283666 25281734

[11] Kastner M, Wilczynski NL, Walker-Dilks C, McKibbon KA, Haynes B (2006). Age-specific search strategies for Medline. J Med Internet Res.

[12] Pang W-K, Leung P-K, Huang W-K, Liu W (2005). On interval estimation of the coefficient of variation for the three-parameter Weibull, lognormal and gamma distribution: a simulation-based approach. Eur J Oper Res.

[13] Hartley R, Green M, Quinn M, Levene M (1993) Pharmacokinetics of morphine infusion in premature neonates. Archives of disease in childhood 69 (1 Spec No):55-58 10.1136/adc.69.1_spec_no.55 PMC1029400 8346956

[14] Mikkelsen S, Feilberg V, Christensen C, Lundstrøm K (1994). Morphine pharmacokinetics in premature and mature newborn infants. Acta Paediatr.

[15] Barrett D, Elias‐Jones A, Rutter N, Shaw P, Davis S (1991). Morphine kinetics after diamorphine infusion in premature neonates. Br J Clin Pharmacol.

[16] Chay PC, Duffy BJ, Walker JS (1992). Pharmacokinetic-pharmacodynamic relationships of morphine in neonates. Clin Pharmacol Ther.

[17] Saarenmaa E, Neuvonen PJ, Rosenberg P, Fellman V (2000). Morphine clearance and effects in newborn infants in relation to gestational age&ast. Clin Pharmacol Ther.

[18] Barrett D, Barker D, Rutter N, Pawula M, Shaw P (1996). Morphine, morphine‐6‐glucuronide and morphine‐3‐glucuronide pharmacokinetics in newborn infants receiving diamorphine infusions. Br J Clin Pharmacol.

[19] Choonara I, McKay P, Hain R, Rane A (1989). Morphine metabolism in children. Br J Clin Pharmacol.

[20] Geiduschek JM, Lynn AM, Bratton SL, Sanders JC, Levy FH, Haberkern CM, O'Rourke PP (1997). Morphine pharmacokinetics during continuous infusion of morphine sulfate for infants receiving extracorporeal membrane oxygenation. Crit Care Med.

[21] Bhat R, Chari G, Gulati A, Aldana O, Velamati R, Bhargava H (1990). Pharmacokinetics of a single dose of morphine in preterm infants during the first week of life. J Pediatr.

[22] Scott CS, Riggs KW, Ling EW, Fitzgerald CE, Hill ML, Grunau RV, Solimano A, Craig KD (1999). Morphine pharmacokinetics and pain assessment in premature newborns. J Pediatr.

[23] Lynn A, Nespeca M, Bratton S, Strauss S, Shen D (1998). Clearance of morphine in postoperative infants during intravenous infusion: the influence of age and surgery. Anesth Analg.

[24] Lynn AM, Slattery JT (1987). Morphine pharmacokinetics in early infancy. Anesthesiology.

[25] McRorie TI, Lynn AM, Nespeca MK, Opheim KE, Slattery JT (1992). The maturation of morphine clearance and metabolism. Arch Pediatr Adolesc Med.

[26] Koren G, Butt W, Chinyanga H, Soldin S, Tan Y-K, Pape K (1985). Postoperative morphine infusion in newborn infants: assessment of disposition characteristics and safety. J Pediatr.

[27] Róka A, Melinda KT, Vásárhelyi B, Machay T, Azzopardi D, Szabó M (2008). Elevated morphine concentrations in neonates treated with morphine and prolonged hypothermia for hypoxic ischemic encephalopathy. Pediatrics.

[28] Olkkola KT, Maunuksela E-L, Korpela R, Rosenberg PH (1988). Kinetics and dynamics of postoperative intravenous morphine in children. Clin Pharmacol Ther.

[29] Choonara I, Lawrence A, Michalkiewicz A, Bowhay A, Ratcliffe J (1992). Morphine metabolism in neonates and infants. Br J Clin Pharmacol.

[30] Lynn AM, Nespeca MK, Bratton SL, Shen DD (2003). Ventilatory effects of morphine infusions in cyanotic versus acyanotic infants after thoracotomy. Pediatr Anesth.

[31] Shelly M, Cory E, Park G (1986). Pharmacokinetics of morphine in two children before and after liver transplantation. Br J Anaesth.

[32] Nahata M, Miser A, Miser J, Reuning R (1985). Variation in morphine pharmacokinetics in children with cancer. Dev Pharmacol Ther.

[33] Collins JJ, Geake J, Grier HE, Houck CS, Thaler HT, Weinstein HJ, Twum-Danso NY, Berde CB (1996). Patient-controlled analgesia for mucositis pain in children: a three-period crossover study comparing morphine and hydromorphone. J Pediatr.

[34] Dampier CD, Setty B, Logan J, Ioli JG, Dean R (1995). Intravenous morphine pharmacokinetics in pediatric patients with sickle cell disease. J Pediatr.

[35] Robie IC, Kellner JD, Coppes MJ, Shaw D, Brown E, Good C, O'brodovich H, Manson D, Olivieri NF, Zipursky A (1992). Analgesia in children with sickle cell crisis: comparison of intermittent opioids vs. continuous intravenous infusion of morphine and placebo-controlled study of oxygen inhalation. Pediatr Hematol-Oncol.

[36] Mashayekhi SO, Ghandforoush‐Sattari M, Routledge PA, Hain RD (2009). Pharmacokinetic and pharmacodynamic study of morphine and morphine 6‐glucuronide after oral and intravenous administration of morphine in children with cancer. Biopharm Drug Dispos.

[37] Hartley R, Quinn M, Green M, Levene M (1993). Morphine glucuronidation in premature neonates. Br J Clin Pharmacol.

[38] Bouwmeester N, Van Den Anker J, Hop W, Anand K, Tibboel D (2003). Age‐and therapy‐related effects on morphine requirements and plasma concentrations of morphine and its metabolites in postoperative infants. Br J Anaesth.

[39] Berkenstadt H, Segal E, Mayan H, Almog S, Rotenberg M, Perel A, Ezra D (1999). The pharmacokinetics of morphine and lidocaine in critically ill patients. Intensive Care Med.

[40] Rudin Å, Lundberg JF, Hammarlund-Udenaes M, Flisberg P, Werner MU (2007). Morphine metabolism after major liver surgery. Anesth Analg.

[41] Bodenham A, Shelly M, Park G (1988). The altered pharmacokinetics and pharmacodynamics of drugs commonly used in critically ill patients. Clin Pharmacokinet.

