# Supplementary material for: Inter-individual variation in morphine clearance in children
Source: Eur J Clin Pharmacol. 2015 Apr 8;71(6):649–55. doi: 10.1007/s00228-015-1843-x (PMC4430598; doi:10.1007/s00228-015-1843-x)
Supplement: Supplementary file 1 — (DOCX 58 kb) [file 228_2015_1843_MOESM1_ESM.docx]

Supplementary Table 1 Morphine doses in all age groups

| Age group | Loading dose or  Single dose (SD)  µg/kg/h | Morphine infusion  µg/kg/h | Number of blood samples collected | Study |
| --- | --- | --- | --- | --- |
| Preterm neonates | 100 | 12.5 | 6 | Hartley et al 1993 [[13](#_ENREF_13)] |
|  | 200 | 50 | 6 |  |
|  | 150 | NA | 5-7 | Mikkelsen et al 1994 [[14](#_ENREF_14)] |
|  | 50 | 15 | 12-15 | Barrett et al 1991 [[15](#_ENREF_15)] |
|  | 10-100 | 7.5-30 | 4-19 | Chay et al 1992 [[16](#_ENREF_16)] |
|  | 140 | 20 | 5 | Saarenmaa et al 2000 [[17](#_ENREF_17)] |
|  | 50 or 200 | 15 | 4-9 | Barrett et al 1996 [[18](#_ENREF_18)] |
|  | NA | 10-40 | NA | Choonara et al 1989 [[19](#_ENREF_19)] |
|  | 100 | 10-20 | 4 | Geiduschek et al 1997 [[20](#_ENREF_20)] |
|  | 100 | 12.5 and 25 | NA | Hartley et al 1993 [[37](#_ENREF_37)] |
|  | 100 | 12.5 and 25 | NA |  |
|  | 200 | 50 | NA |  |
|  | 100 | NA | 8 | Bhat et al 1990 [[21](#_ENREF_21)] |
|  | 50 | 20-30 | 6 | Scott et al 1999 [[22](#_ENREF_22)] |
|  | 100 | 12.5-25 | NA | Hartley et al 1993 [[37](#_ENREF_37)] |
| Term neonates | NA | 11 | 2 | Lynn et al 1998 [[23](#_ENREF_23)] |
|  | 100 | 10-30 | 4 | Bouwmeester et al 2003 [[38](#_ENREF_38)] |
|  | NA | 20-100 | 11 | Lynn et al 1987 [[24](#_ENREF_24)] |
|  | NA | 20 | 10 | McRorie et al 1992 [[25](#_ENREF_25)] |
|  | 150 | NA | 5-7 | Mikkelsen et al 1994 [[14](#_ENREF_14)] |
|  | 10-100 | 7.5-30 | 4-19 | Chay et al 1992 [[16](#_ENREF_16)] |
|  | 100 | NA | 14 | Pokela et al 1993 [[5](#_ENREF_5)] |
|  | NA | 10-40 | 1 | Choonara et al 1992 [[29](#_ENREF_29)] |
|  | 50-100 | 6-40 | 3-5 | Koren et al 1985 [[26](#_ENREF_26)] |
|  | 100 | NA | 8 | Bhat et al 1990 [[21](#_ENREF_21)] |
|  | 50 | 20-30 | 6 | Scott et al 1999 [[22](#_ENREF_22)] |
| Infants | 100 | NA | 14 | Pokela et al 1993 [[5](#_ENREF_5)] |
|  | 50 | 25-35 | 2 | Lynn et al 1998 [[23](#_ENREF_23)] |
|  | 50-150 | 5-30 | 5 | Roka et al 2008 [[27](#_ENREF_27)] |
|  | NA | 50 | 11 | Olkkola et al 1988 [[28](#_ENREF_28)] |
|  | NA | 20-100 | 11 | Lynn et al 1987 [[24](#_ENREF_24)] |
|  | NA | 8-47 | 1 | Choonara et al 1992 [[29](#_ENREF_29)] |
|  | NA | 30 | 10 | McRorie et al 1992 [[25](#_ENREF_25)] |
|  | 50 (x2) | 11-19 | 2 | Lynn et al 2003 [[30](#_ENREF_30)] |
|  | 100 | 10-30 | 4 | Bouwmeester et al 2003 [[38](#_ENREF_38)] |
| 2-11 years | 1000 | NA | 9 | Shelly et al 1986 [[31](#_ENREF_31)] |
|  | NA | 15-140 | NA | Choonara et al 1989 [[19](#_ENREF_19)] |
|  | 90-150 | NA | 3 | Nahata et al 1985 [[32](#_ENREF_32)] |
|  | NA | 50 | 11 | Olkkola et al 1988 [[28](#_ENREF_28)] |
| 12-18 years | NA | 15-140 | NA | Choonara et al 1989 [[19](#_ENREF_19)] |
|  | 90-150 | NA | 3 | Nahata et al 1985 [[32](#_ENREF_32)] |
| 1-15yrs | 28-100 | 10 | 3 | Collins et al 1996 [[33](#_ENREF_33)] |
| 6-19 years | NA | 35-195 | 13 | Dampier et al 1995 [[34](#_ENREF_34)] |
| 3-18years | 150 | 40 | NA | Robie et al 1992 [[35](#_ENREF_35)] |
| 2.5-16 years | NA | 150-280 | 2 | Mashayekhi et al 2009 [[36](#_ENREF_36)] |
| 7months-7years | NA | 20-40 | 5 | Dagan et al 1993 [[7](#_ENREF_7)] |

NA, not available

Supplementary Table 2 Range and variation ratio of critically ill patients in relation to M6G/M

| Number of patients | Mean plasma concentration  (ng mlˉ¹)  Morphine M6G | | Mean Plasma  M6G/M | Range  (M6G/M) | Variation  Ratio  (M6G/M) | Dose µg/kg/h | Study |
| --- | --- | --- | --- | --- | --- | --- | --- |
| Neonates | | | | | | | |
| 52 | NA | NA | NA | 0.5-2 | 4 | 10-30 | Bouwmeester et al 2003 [[38](#_ENREF_38)] |
| 8 | 108 | 55 | 0.7 | 1.4-46 | 33 | 50 | Barrett et al 1996 [[18](#_ENREF_18)] |
| 11 | 70 | 44 | 0.9 | 0.1-3 | 30 | 200 |  |
| Infants (>28 days-23 months) | | | | | | | |
| 97 | NA | NA | NA | 0.5-6 | 12 | 10-30 | Bouwmeester et al 2003 [[38](#_ENREF_38)] |
| 1-15years | | | | | | | |
| 10 | NA | NA | 2 | 1-3 | 3 | 28-100 | Collins et al 1996 [[33](#_ENREF_33)] |

NA; not available

Supplementary Table 3 Quality assessment of morphine PK studies in paediatric

| Study | Age group | *Number of patients | +Number of blood samples collected | Method | Verdict |
| --- | --- | --- | --- | --- | --- |
| Hartley et al 1993 [[13](#_ENREF_13)] | Preterm neonates | 9 | 6 | Non compartment | Included |
| Mikkelsen et al 1994 [[14](#_ENREF_14)] |  | 8 | 5-7 | Non compartment | Included |
| Barrett et al 1991 [[15](#_ENREF_15)] |  | 26 | 12-15 | Non compartment | Included |
| Chay et al 1992 [[16](#_ENREF_16)] |  | 10 | 4-19 | Non compartment | Included |
| Saarenmaa et al 2000 [[17](#_ENREF_17)] |  | 31 | 5 | Non compartment | Included |
| Barrett et al 1996 [[18](#_ENREF_18)] |  | 19 | 4-9 | Non compartment | Included |
| Choonara et al 1989 [[19](#_ENREF_19)] |  | 9 | NA | Non compartment | Excluded |
| Geiduschek et al 1997 [[20](#_ENREF_20)] |  | 11 | 4 | Non compartment | Included |
| Hartley et al 1993 [[37](#_ENREF_37)] |  | 10 | NA | Non compartment | Excluded |
| Bhat et al 1990 [[21](#_ENREF_21)] |  | 10  7 | 8 | Non compartment | Included |
| Scott et al 1999 [[22](#_ENREF_22)] |  | 9  13  13 | 6 | Non compartment | Included |
| Lynn et al 1998 [[23](#_ENREF_23)] | Term neonates | 4 | 2 | Non compartment | Excluded |
| Bouwmeester et al 2003 [[38](#_ENREF_38)] |  | 52 | 4 | Non compartment | Included |
| Lynn et al 1987 [[24](#_ENREF_24)] |  | 6 | 11 | Non compartment | Included |
| McRorie et al 1992 [[25](#_ENREF_25)] |  | 18 | 10 | Non compartment | Included |
| Mikkelsen et al 1994 [[14](#_ENREF_14)] |  | 5 | 5-7 | Non compartment | Included |
| Chay et al 1992 [[16](#_ENREF_16)] |  | 5 | 4-19 | Non compartment | Included |
| Choonara et al 1992 [[29](#_ENREF_29)] |  | 6 | 1 | Non compartment | Excluded |
| Koren et al 1985 [[26](#_ENREF_26)] |  | 12 | 3-5 | Non compartment | Included |
| Bhat et al 1990 [[21](#_ENREF_21)] |  | 3 | 8 | Non compartment | Included |
| Scott et al 1999 [[22](#_ENREF_22)] |  | 3 | 6 | Non compartment | Included |
| Pokela et al 1993 [[5](#_ENREF_5)] |  | 10  10 | 14 | Non compartment | Included |
| Lynn et al 1998 [[23](#_ENREF_23)] | Infants | 22 | 2 | Non compartment | Excluded |
| Olkkola et al 1988 [[28](#_ENREF_28)] |  | 5 | 11 | Non compartment | Included |
| Choonara et al 1992 [[29](#_ENREF_29)] |  | 2 | 1 | Non compartment | Excluded |
| Lynn et al 1987 [[24](#_ENREF_24)] |  | 3 | 11 | Non compartment | Included |
| Bouwmeester et al 2003 [[38](#_ENREF_38)] |  | 97 | 4 | Non compartment | Included |
| McRorie et al 1992 [[25](#_ENREF_25)] |  | 35 | 10 | Non compartment | Included |
| Pokela et al 1993 [[5](#_ENREF_5)] |  | 2 | 14 | Non compartment | Excluded |
|  |  | 5 |  | Non compartment | Included |
| Roka et al 2008 [[27](#_ENREF_27)] |  | 10  6 | 5 | Non compartment | Included |
| Lynn et al 2003 [[30](#_ENREF_30)] |  | 10  10 | 2 | Non compartment | Excluded |
| Shelly et al 1986 [[31](#_ENREF_31)] | 2-11 years | 2 | 9 | Non compartment | Excluded |
| Choonara et al 1989 [[19](#_ENREF_19)] |  | 4 | NA | Non compartment | Excluded |
| Nahata et al 1985 [[32](#_ENREF_32)] |  | 3 | 3 | Non compartment | Included |
| Olkkola et al 1988 [[28](#_ENREF_28)] |  | 9 | 11 | Non compartment | Included |
| Choonara et al 1989 [[19](#_ENREF_19)] | 12-18 years | 4 | NA | Non compartment | Excluded |
| Nahata et al 1985 [[32](#_ENREF_32)] |  | 3 | 3 | Non compartment | Included |
| Collins et al 1996 [[33](#_ENREF_33)] | 1-15yrs | 10 | 3 | Non compartment | Included |
| Dampier et al 1995 [[34](#_ENREF_34)] | 6-19 years | 18 | 13 | Two compartment | Included |
| Robie et al 1992 [[35](#_ENREF_35)] | 3-18years | 24 | NA | Non compartment | Excluded |
| Mashayekhi et al 2009 [[36](#_ENREF_36)] | 2.5-16 years | 7 | 2 | One compartment | Excluded |
| Dagan et al 1993 [[7](#_ENREF_7)] | 7months-7years | 14  23 | 5 | Non compartment | Included |

* Studies with **≤** 2 patients or with number of patients not stated are excluded, ^+^ Studies with **≤**2 samples or with the number of samples not stated are excluded, NA, not available
